# Supplementary material for: The Safety Assessment of Mutagenicity, Acute and Chronic Toxicity of the Litsea martabanica (Kurz) Hook.f. Water Leaf Extract
Source: Toxics. 2024 Jun 28;12(7):470. doi: 10.3390/toxics12070470 (PMC11281209; doi:10.3390/toxics12070470)
Supplement: Supplementary file 1 [file toxics-12-00470-s001.zip › toxics-3009033-supplementary.pdf]

Supplementary Data

The Safety Assessment of Mutagenicity, Acute and ChronicToxicity of the *Litsea martabanica* (Kurz) Hook.f. Water Leaf Extract

Table S1. Hippocratic screening for acute toxicity of *L. martabanica* leaf extract in female rats.

***L. martabanica* leaf extract (mg/kg)**	**Hours after drug administration**																							
**5,000**	1	2	3	4	5	6	7	8	9	10	11	12	13	14	15	16	17	18	19	20	21	22	23	24
Decrease of motor activity	0	0	0	0	0	0	0	0	0	0	0	0	0	0	0	0	0	0	0	0	0	0	0	0
Decrease of respiratory rate	0	0	0	0	0	0	0	0	0	0	0	0	0	0	0	0	0	0	0	0	0	0	0	0
Loss of righting reflex	0	0	0	0	0	0	0	0	0	0	0	0	0	0	0	0	0	0	0	0	0	0	0	0
Loss of screen grip	0	0	0	0	0	0	0	0	0	0	0	0	0	0	0	0	0	0	0	0	0	0	0	0
Time of death (hr.)	–	–	–	–	–	–	–	–	–	–	–	–	–	–	–	–	–	–	–	–	–	–	–	–

Decrease in motor activity: 0 = no decrease in motor activity, no change in respiratory rate, no loss of righting reflex, no loss of screen grip; +1 = does not move spontaneously, but when handled will move rapidly; +2 = when handled will move slowly; +3 = when handled will move sluggishly; +4 = when handled will not move at all. Decrease in respiration rate: +1 = 10% decrease in respiratory rate; +2 = 20% decrease in respiratory rate; +3 = 40% decrease in respiratory rate; +4 = 80% decrease in respiratory rate. Loss of righting reflex: +1 = can be placed only on one side; +2 = can be placed on either side equally well; +3 = can be placed on back as well as either side; +4 = cannot be aroused from back position by the hind leg toe pinch. Loss of screen grip: +1 = rat falls off at first shake of screen; +2 = rat falls off when screen has been inverted; +3 = rat falls off when the screen is at a 90° angle; +4 = rat falls off as the screen is tilted to a 45° angle.

Table S2. Hippocratic screening for chronic toxicity of *L. martabanica* leaf extract in both female and male rats.

***L. martabanica* leaf extract (mg/kg) 250, 750, 2,250, 2,250 Satellite**	**Weeks after drug administration**																				
	1-2	3-4	5-6	7-8	9-10	11-12	13-14	15-16	17-18	19-20	21-22	23-24	25-26	27-28	29-30	31-32	33-34	35-36	37-38	39-40	41-42
Decrease of motor activity	0	0	0	0	0	0	0	0	0	0	0	0	0	0	0	0	0	0	0	0	0
Decrease of respiratory rate	0	0	0	0	0	0	0	0	0	0	0	0	0	0	0	0	0	0	0	0	0
Loss of righting reflex	0	0	0	0	0	0	0	0	0	0	0	0	0	0	0	0	0	0	0	0	0
Loss of screen grip	0	0	0	0	0	0	0	0	0	0	0	0	0	0	0	0	0	0	0	0	0
Time of death (wk.)	–	–	–	–	–	–	–	–	–	–	–	–	–	–	–	–	–	–	–	–	–

Decrease in motor activity: 0 = no decrease in motor activity, no change in respiratory rate, no loss of righting reflex, no loss of screen grip; +1 = does not move spontaneously, but when handled will move rapidly; +2 = when handled will move slowly; +3 = when handled will move sluggishly; +4 = when handled will not move at all. Decrease in respiration rate: +1 = 10% decrease in respiratory rate; +2 = 20% decrease in respiratory rate; +3 = 40% decrease in respiratory rate; +4 = 80% decrease in respiratory rate. Loss of righting reflex: +1 = can be placed only on one side; +2 = can be placed on either side equally well; +3 = can be placed on back as well as either side; +4 = cannot be aroused from back position by the hind leg toe pinch. Loss of screen grip: +1 = rat falls off at first shake of screen; +2 = rat falls off when screen has been inverted; +3 = rat falls off when the screen is at a 90° angle; +4 = rat falls off as the screen is tilted to a 45° angle.
